# Complementary/Alternative Medicine in Cardiovascular Diseases 2014

**DOI:** 10.1155/2015/349375

**Published:** 2015-05-20

**Authors:** Ke-Ji Chen, Myeong Soo Lee, Hao Xu, Qunhao Zhang

**Affiliations:** ^1^Cardiovascular Center, Xiyuan Hospital, China Academy of Chinese Medical Sciences, Beijing 100091, China; ^2^Clinical Research Division, Korea Institute of Oriental Medicine, Daejeon 305-811, Republic of Korea; ^3^Department of Medicine, Massachusetts General Hospital, Harvard Medical School, Boston, MA 02114, USA

Cardiovascular diseases (CVDs) are the leading cause of long-term morbidity and mortality worldwide and are on an alarming rise in populations. According to the report of World Health Organization (WHO) in 2012, an estimated 17.5 million people died of CVDs, accounting for 31% of global deaths. As the increasing significance is attached to CVDs globally, the urgency to respond to CVDs with stronger and more effective therapies is widely recognized. Despite enormous efforts, revascularization, and secondary prevention, to prevent CVDs in the past, major challenges remain to cope with repeated recurrent acute cardiovascular events, readmission to the hospital and improvement of the long-term prognosis and quality of life. In this case, complementary and alternative medicine (CAM) therapies have the potential to provide a major public health benefit.

In this special issue, papers from different parts of the world like China, Republic of Korea, Malaysia, Columbia, Chile, and so forth, are presented. These articles provide a review of this field and make original contributions towards the mechanism of action and the clinical application of CAM for CVDs. In these studies, antiatherogenesis effects of some CAM products were highlighted, including the extracts of herbal plants such as paeonol (Pae), polysaccharide of* Polygonatum sibiricum* (PPGS), or fermented mung bean and fermented red yeast rice. Their cardioprotective role in hypolipidemic, antioxidant, and antiatherogenesis was introduced. In addition, a study on the consumption of apple peel with rich phenolic compounds concluded that it reduced several metabolic syndrome parameters and the atherogenic progression in mice.

Attention was also paid to treatment of ischemia/reperfusion (I/R) injury. Increasing evidence has indicated that traditional Chinese medicine (TCM) could significantly prevent myocardial apoptosis and provide alternative options for protection of myocardial I/R injury.* Huangzhi oral liquid* was an effective treatment for arrhythmias by increasing caspase-3 and apoptosis network proteins.* Shuang Shen Ning Xin* played an important antiapoptotic role by blocking the mitochondrial apoptotic pathway. Neuroprotection of* Sanhua decoction* against focal cerebral ischemia/reperfusion injury was demonstrated in rats through a mechanism targeting aquaporin 4. Two papers about ginsenoside Rb1 (GS-Rb1) discussed how to protect hypoxia- and ischemia-induced cardiomyocytes by regulating expression of miRNAs and inhibition of the mitochondrial apoptotic pathway, respectively.

The roles of Chinese medicine and other CAM therapies in relieving symptoms of hypertension, angina pectoris, and chronic heart failure were reviewed in several articles. A systematic review of randomized controlled trials showed that Chinese herbal medicine (CHM) combined with conventional therapies was effective in controlling blood pressure variability and symptoms of hypertension. Two other papers also highlighted the roles of integrative medicine therapy, for instance,* Xuefu Zhuyu decoction* functioning together with traditional antianginal medications could relieve the clinical symptoms of angina pectoris;* Wenxin Keli* and sotalol could effectively facilitate sinus rhythm reversion from hyperthyroidism related paroxysmal atrial fibrillation. In addition, the advantage of* Dangguijagyagsan*, a Korean traditional herbal prescription, on the treatment for cardiovascular diseases of menopausal women was referred to in a study. Another three-stage, multicenter, clinical trial evaluated the efficacy, safety, feasibility, compliance, and universality of CHM in the treatment of chronic heart failure.

A variety of functional readouts of CAM may reveal new therapeutic strategies to manipulate cardioprotection, which could be transferred into application of treating CVDs. However, despite the growing utility of CAM, rigorous evidence-based studies with high quality are required to reinforce the application of CAM among CVD patients, especially its clinical benefit, prognostic impact, and potential interaction when used in combination with prescription medicines. It is widely acknowledged that there should be great potential in developing CAM use based on open dialogue between mainstream medicine doctors and CAM practitioners, healthcare professionals and patients.

*Ke-Ji Chen* *Ke-Ji Chen* *Myeong Soo Lee* *Myeong Soo Lee* *Hao Xu* *Hao Xu* *Qunhao Zhang* *Qunhao Zhang*

